# A *COL4A1* variant in a neonate with multiple intracranial hemorrhages and congenital cataracts

**DOI:** 10.1038/s41439-022-00199-5

**Published:** 2022-06-10

**Authors:** Ayane Yakabe, Tamaki Ikuse, Natsuki Ito, Hiromichi Yamada, Nobutomo Saito, Yuri Kitamura, Tomohiro Iwasaki, Mitsuru Ikeno, Hiroki Suganuma, Shinpei Abe, Nao Miyazaki, Ken Hisata, Hiromichi Shoji, Tomoyuki Nakazawa, Hidetaka Eguchi, Toshiaki Shimizu

**Affiliations:** 1 Department of Pediatrics, Juntendo University Faculty of Medicine, Tokyo, Japan; 2 Diagnostics and Therapeutics of Intractable Diseases and Intractable Disease Research Center, Juntendo University Graduate School of Medicine, Tokyo, Japan

**Keywords:** Disease genetics, Cerebrovascular disorders

## Abstract

A 2-day-old neonate presented with seizures, multiple intracranial hemorrhages, and bilateral congenital cataracts. Targeted next-generation sequencing of the collagen type IV alpha 1 chain (*COL4A1*) gene revealed a heterozygous de novo missense variant (NM_001845.6:c.2291G>A/p.Gly764Asp). This missense variant adds to the compendium of *COL4A1* variants and is associated with a *COL4A1*-related disorder.

Type IV collagen (COL4) is the most predominant and widely expressed protein in the basement membranes of systemic organ tissues. It has a helical heterotrimeric structure formed by three α-chain sets of α1-α6 and mainly consists of α1 and combinations of α1 and α2. The *COL4A1* and *COL4A2* genes reside on chromosome 13q, encoding the α1 and α2 chains of COL4, respectively. It is well known that pathogenic variations in *COL4A1/A2* cause abnormalities in the basement membranes of systemic organs. For example, various cerebrovascular diseases, such as the development of porencephaly, schizencephaly, intracerebral hemorrhage, periventricular hyperintensity, ventricular enlargement, cerebellar atrophy, intracerebral calcification, cerebral infarction, and cerebral aneurysm, caused by the fragility of the basement membranes of cerebral blood vessels have been reported. Less lethal complications include cataracts, microphthalmia, tortuous retinal arterioles, retinal hemorrhages, hematuria, proteinuria, renal dysfunction, renal cysts, arrhythmia, elevated serum creatine kinase levels, muscle spasms, Raynaud’s phenomenon, and hemolytic anemia^1^. Most *COL4A1/A2* variants result from missense variants due to the substitution of glycine in the Gly-Xaa-Yaa triple-helical domain^2^. In addition, splice-site mutations leading to haploinsufficiency and frameshift mutations have been reported, indicating that the haploinsufficiency of either *COL4A1* or *COL4A2* is another pathogenic mechanism^3^. The resulting phenotype and severity vary from case to case, and neurological and other prognoses differ greatly depending on the severity of complications. Throughout life, the risk of cerebral hemorrhage is higher than that in the normal population, and the risk of bleeding is further increased by exercise, surgical intervention, and anticoagulant medication use. Investigations of treatments have been limited to nonclinical studies; therefore, no effective treatment has been established for this disorder.

We report the case of a 2-day-old male neonate who was born healthy at 36 weeks and 4 days of gestation with normal vaginal delivery in another hospital, with a birth weight of 2606 g (+0.14 standard deviation) and height of 46.6 cm (−0.11 standard deviation). On the second postnatal day, the patient presented with a generalized clonic seizure. Brain ultrasonography and computed tomography (CT) scans were suggestive of multiple intracranial hemorrhages with intraventricular perforation. On Day 3, the patient was transferred to our hospital (Juntendo University Hospital) for the management and treatment of multiple intracranial hemorrhages and seizures. A magnetic resonance imaging (MRI) scan revealed multiple parenchymal and subdural hemorrhages and dilation of the blood vessels in the semioval center (Fig. 1). In contrast, there were no signs of tumor, blood vessel dissection, or venous sinus thrombosis. The presence of cerebrovascular disease and bilateral congenital cataracts, revealed by ophthalmological evaluation, led us to suspect a *COL4A1/A2*-related disorder.

**Fig. 1:**
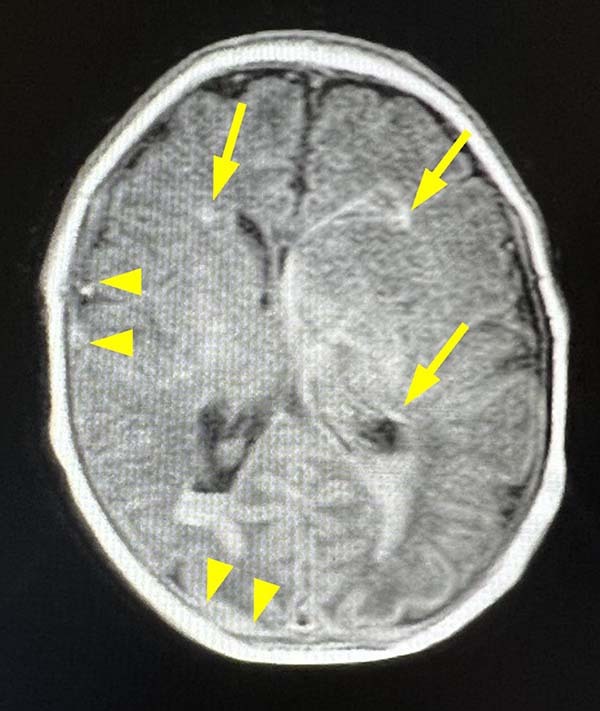
Magnetic resonance imaging (MRI) scan of the brain. Axial view, T1-weighted images show multiple parenchymal hemorrhages (arrows) and subdural hemorrhages (arrowheads).

The patient was the first child of nonconsanguineous parents with no family history of cerebral hemorrhages, seizures, cataracts, or other diseases associated with *COL4A1/A2*-related disorders. Chromosome karyotyping using the G-banding technique revealed a 46, XY male karyotype. The hybridization capture method for targeted next-generation sequencing of the *COL4A1/A2* gene was performed with informed consent from the parents. A heterozygous missense variant NM_001845.6:c.2291G>A/p. Gly764Asp of the *COL4A1* gene was identified in the patient’s DNA extracted from his blood sample. After obtaining informed consent, the DNA of the patient and his parents was analyzed using Sanger sequencing. The missense variant was identified only in the patient’s blood DNA, and there were no variants in the parents’ DNA (Fig. 2).

**Fig. 2:**
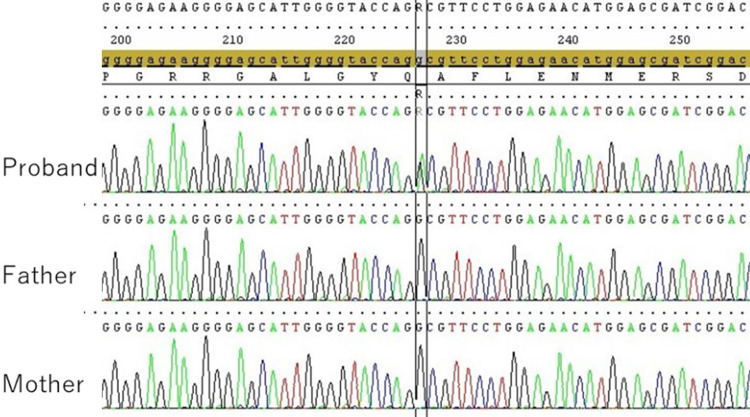
Detection of the *COL4A1* variant by Sanger sequencing. Sanger sequencing revealed a heterozygous missense variant (NM_001845.6:c.2291G>A) in the proband’s DNA (top) but not in the father’s (middle) or mother’s (bottom) blood DNA.

The results of genome sequencing and clinical findings were further assessed using the ACMG/AMP guidelines^4^ for the interpretation of sequence variants. The *COL4A1* variant of the patient possessed a de novo mutation (PS2). The identified missense variant leads to a substitution of a glycine in the Gly-X-Y repeats in the triple-helical domain of the *COL4A1* gene; a number of pathogenic variants have accumulated substitutions in this Gly-X-Y repeat (PM1)^3^. The variant is extremely rare and is not listed in gnomAD (https://gnomad.broadinstitute.org/), an international genome database for healthy individuals, or jMorp 14KJPN (https://jmorp.megabank.tohoku.ac.jp/202112/variants/), a genome database for healthy Japanese individuals (PM2). Structural predictions using Polyphen-2, SIFT, and PROVEAN resulted in deleterious/damaging changes (PP3). In addition, the symptoms observed in the patient were typical of a *COL4A1*-related disorder (PP4). Moreover, the diagnosis of a *COL4A1*-related disorder was confirmed based on molecular investigations. Considering all these findings, the variant was classified as pathogenic in the context of *COL4A1*-related disorders (PS2, PM1, PM2, PP3, PP4) according to the ACMG/AMP guidelines. To our knowledge, the variant has not been described previously and is not present in the Human Gene Mutation Database Professional (2021.10). It was registered as pathogenic by Athena Diagnostics Inc. in ClinVar (http://www.clinvar.com) without functional evidence.

Although *COL4A1/A2*-related disorders have an autosomal dominant inheritance, 30% of the cases occur de novo. Therefore, sequencing of the *COL4A1/A2* gene is recommended for neonates with porencephaly, schizencephaly, or cerebral microvascular lesions, regardless of the family history of *COL4A1/A2*-related disorders^3^. In this case, multiple intracranial hemorrhages experienced during the patient’s course of birth and the presence of bilateral congenital cataracts led us to perform molecular testing to identify the *COL4A1/A2* variant, and we diagnosed the patient with a *COL4A1*-related disorder. Since the variant was not detected in the parents’ blood DNA, the risk of the disorder in future siblings could be considered low. However, future pregnancies should be carefully monitored because genome sequencing was performed only against blood DNA and not against tissue DNA.

In conclusion, we identified a de novo missense variant of the *COL4A1* gene in a neonate leading to a *COL4A1*-related disorder manifesting as multiple intracranial hemorrhages and bilateral congenital cataracts.

## Data Availability

The relevant data from this Data Report are hosted at the Human Genome Variation Database at 10.6084/m9.figshare.hgv.3202.
